# Genomic Regions Associated with Resistance to Gastrointestinal Parasites in Australian Merino Sheep

**DOI:** 10.3390/genes15070846

**Published:** 2024-06-27

**Authors:** Brenda Vera, Elly A. Navajas, Pablo Peraza, Beatriz Carracelas, Elize Van Lier, Gabriel Ciappesoni

**Affiliations:** 1 Sistema Ganadero Extensivo, INIA Las Brujas, Canelones 90200, Uruguay; bvera@inia.org.uy (B.V.); enavajas@inia.org.uy (E.A.N.); peraza@inia.org.uy (P.P.); bcarracelas@inia.org.uy (B.C.); 2 Departamento de Producción Animal y Pasturas, Facultad de Agronomía, Universidad de la República, Avda. Garzón 780, Montevideo 12900, Uruguay; evanlier@fagro.edu.uy; 3 Estación Experimental Facultad de Agronomía Salto, Salto 50000, Uruguay

**Keywords:** ssGBLUP, ssGWAS, *Ovis aries*, *Haemonchus contortus*, nematodes

## Abstract

The objective of this study was to identify genomic regions and genes associated with resistance to gastrointestinal nematodes in Australian Merino sheep in Uruguay, using the single-step GWAS methodology (ssGWAS), which is based on genomic estimated breeding values (GEBVs) obtained from a combination of pedigree, genomic, and phenotypic data. This methodology converts GEBVs into SNP effects. The analysis included 26,638 animals with fecal egg count (FEC) records obtained in two independent parasitic cycles (FEC1 and FEC2) and 1700 50K SNP genotypes. The comparison of genomic regions was based on genetic variances (gVar(%)) explained by non-overlapping regions of 20 SNPs. For FEC1 and FEC2, 18 and 22 genomic windows exceeded the significance threshold (gVar(%) ≥ 0.22%), respectively. The genomic regions with strong associations with FEC1 were located on chromosomes OAR 2, 6, 11, 21, and 25, and for FEC2 on OAR 5, 6, and 11. The proportion of genetic variance attributed to the top windows was 0.83% and 1.9% for FEC1 and FEC2, respectively. The 33 candidate genes shared between the two traits were subjected to enrichment analysis, revealing a marked enrichment in biological processes related to immune system functions. These results contribute to the understanding of the genetics underlying gastrointestinal parasite resistance and its implications for other productive and welfare traits in animal breeding programs.

## 1. Introduction

The Australian Merino breed is a wool breed traditionally bred in Uruguay in extensive production systems, mainly located in the basalt region. Currently, this breed represents about 40% of the country’s sheep stock and is the main breed in fine and superfine wool production. One of the main problems affecting sheep production is infection by gastrointestinal parasites (GIPs) [1]. The decrease in production is a consequence of growth retardation, decreased weight gain, fleece weight and wool quality, and increased mortality [2,3], with *Haemonchus contortus* and *Trichostrongylus colubriformis* being the most prevalent parasites in Uruguay [4].

Given the problem of parasitism and the reported anthelmintic resistance [5,6,7], one alternative control method involves the breeding of animals genetically resistant to GIPs. The selection criterion used to assess resistance is the parasite fecal egg count (FEC), which is a moderately heritable trait [8] included in the National Genetic Evaluations (NGEs) in Uruguay. Currently, the NGE protocol includes the recording of two counts: the initial FEC (FEC1) shortly after weaning (7–9 months of age) and the subsequent FEC (FEC2) at 10–14 months of age [3].

Genome-wide association studies (GWASs) have been implemented in sheep populations to identify genes potentially associated with GIP resistance [9,10,11,12,13]. Unlike other methodologies that are limited to using genomic information exclusively from phenotyped animals, ssGWAS integrates genomic, pedigree, and phenotypic data from both genotyped and non-genotyped animals [14], thus considering population structure [15]. The ssGWAS procedure combines traditional pedigree relationships with those derived from genetic markers, and by the conversion of GEBVs to marker effects and weights [14]. 

The objectives of our study were as follows: (1) to estimate variance and heritability components for FEC1 and FEC2 traits in the Australian Merino sheep population in Uruguay; (2) to identify genomic regions and candidate genes associated with each trait by ssGWAS; and (3) to explore genes associated with FEC1 and FEC2, providing insight into the biological mechanisms underlying resistance to GIPs in sheep.

## 2. Materials and Methods

### 2.1. Natural Parasite Challenge

FEC determination was conducted according to the current protocol used for recording phenotypes in the NGEs [3]. In short, between 8 and 14 days after deworming at weaning, fecal samples were collected randomly from 15 to 20 lambs to assure the efficacy of the anthelmintic and that the animals started the evaluation period with 0 FECs. Every 14 to 21 days, 15 to 20 random lambs of each contemporary group (year, farm, sex) were sampled for FEC to evaluate the progress of the GIN infestation. When the mean of the FEC reached 500, and samples with 0 FECs represented less than 20% of the samples, all individuals were sampled for FEC1. After FEC1, lambs were again dewormed, and the process started over until all individuals were sampled for FEC2. All groups were managed under the same protocol, with lambs receiving oral drenching using proven anthelmintics such as Startec^®^ (Zoetis, Auckland, New Zealand) and TritomNF^®^ (Cibeles, Canelones, Uruguay). 

**Figure 1:**
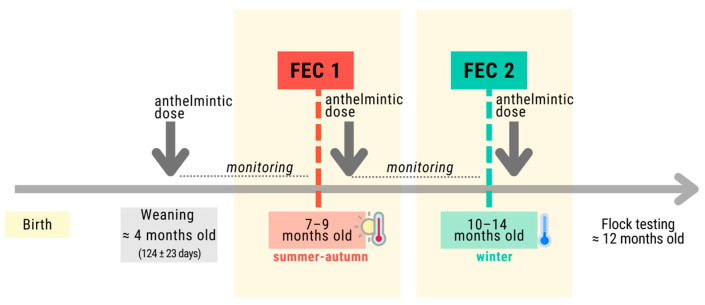
Sampling scheme for fecal egg counts (FECs) in two independent parasitic cycles and in different seasons.

The animals were probably exposed to multiple GIN species on pasture, as was observed in other studies [16]. The specific quantity of parasites ingested by each lamb could not be determined, but fecal sampling occurred during the warmer season (summer/fall) when environmental conditions were favorable for *H. contortus* and during the winter for *Trichostrongylus* sp. (Figure 1). In 2020, an analysis combining coproculture and (q)PCR was performed for the identification of GIN in grazing animals in Uruguay, and the results revealed a higher prevalence of *H. contortus* and *Trichostrongylus* sp. [17]. Previous studies report a prevalence of 43% of *H. contortus* and 38% of *Trichostrongylus* sp. [4]. Therefore, fecal samples were collected from two independent natural parasitic cycles separated by an anthelmintic treatment (Figure 1). Deworming of lambs at weaning and after FEC1 is essential to establish a baseline for comparison among individuals within a contemporary group.

### 2.2. Phenotypic Data

Samples were collected post-weaning; the mean age at recording (days) for FEC1 and FEC2 was 273 (±69) and 341 (±62), respectively. The mean time between each anthelmintic dose and sampling (days) for FEC1 (145 ± 64) and FEC2 (85 ± 40) exceeds the persistence period of anthelmintics in animals (≈15 days). A fecal sample was obtained from each individual directly from the rectum, and the modified McMaster technique with a sensitivity of 100 eggs per gram of feces was used to estimate the FEC [18]. Counts were transformed to natural logarithm, as described by Ciappesoni et al. [8], due to their non-normal distribution (LogFEC = Log_e_ (FEC + 100)). In this study, we refer to logFEC1 and logFEC2 as FEC1 and FEC2, respectively.

A total of 26,638 animals born between 2001 and 2020 and belonging to 13 farms had FEC1 records. Among these, 18,971 animals also had FEC2 records (Table 1).

### 2.3. Genomic and Pedigree Data

Genomic DNA was extracted from blood samples, as described by Carracelas et al. [10]. A total of 1702 individuals were genotyped with the GeneSeek^®^ Genomic Profiler™ (GGP, 43,705 SNPs) BeadChip (GeneSeek, Lincoln, NE, USA). Genomic data quality control was performed using preGSf90 (Aguilar et al., 2014). SNPs with a call rate below 90%, with minor allele frequency (MAF) less than 1%, monomorphic SNPs, and animals with call rate less than 90% were removed. Finally, 38,268 SNPs for 1697 sheep were used in the analysis.

The pedigree file was corrected using SeekParentF90 [19], which detects incompatibilities based on Mendelian conflict counts, as described in Wiggans et al. [20]. 

### 2.4. Statistical Analysis

Genetic parameters were estimated using methods based on pedigree relationships (BLUP) and pedigree–genomic models (ssGBLUP) [21]. A univariate model was conducted to estimate the variance components and heritabilities for FEC1 and FEC2 employing the AIREMLF90 software from the BLUPF90 family of programs [19]. Additionally, genetic correlations between FEC1 and FEC2 were estimated. 

The following univariate model was used:

y=Xb+Zu+e

where *y* is the vector of phenotypes for FEC1 or FEC2; *X* and *Z* are incidence matrices for fixed and random effects, respectively; *b* is the vector of fixed effects, including 494 contemporary groups (year of birth, sex, flock), dam age (three levels: 2, 3, and >4 years), type of birth (two levels: single or multiple), and lamb age at FEC1 or FEC2 recording as a covariate; *u* is the vector of random additive genetic effects; and *e* is the vector of residual effects.

In BLUP estimates, the random effects were modeled as *u*~*N*(0, *A*$\sigma_{a}^{2}$) and *e*~*N*(0, *I*$\sigma_{e}^{2}$), where A represents the numerator relationship matrix, I is the identity matrix, $\sigma_{a}^{2}$ stands for the additive genetic variance, and $\sigma_{e}^{2}$ is the residual variance. In the ssGBLUP model, the numerator relationship matrix ($A^{-1}$) utilized in BLUP is substituted with the $H^{-1}$ matrix.

$$H^{-1}=A^{-1}+\left[\begin{array}{cc} 0 & 0\\ 0 & G^{-1}-A_{22}^{-1} \end{array}\right]$$

where $A^{-1}$ and $A_{22}^{-1}$ are the inverse of the pedigree relationship matrix for all animals and for genotyped animals, respectively, and $G^{-1}$ is the inverse of the genomic relationship matrix. The matrix G was constructed as described by Van Raden [22]:

$$G=ZDZ^{\prime }q$$

where Z is the SNP incidence matrix adjusted for allele frequencies, D is a weight matrix for SNP (initially *D = I*), and q is a weighting factor. The SNP effects and weighting factor were derived using an iterative process described by Wang et al. [14]. In this study, a single iteration was used as there was no significant change in SNP effects with additional iterations.

The percentage of genetic variance explained by region was calculated as follows:

$$\frac{Var(a_{i})}{\sigma_{a}^{2}}\times 100\%=\frac{Var\left(\Sigma_{j=1}^{20}Z_{j}{\hat{u}}_{j}\right)}{\sigma_{a}^{2}}\times 100\%$$

where $a_{i}$ is the genetic value of the *i*-th region (20 contiguous SNPs), $\sigma_{a}^{2}$ is the total genetic variance, $Z_{j}$ is the gene content vector of the *j*-th SNP for all individuals, and ${\hat{u}}_{j}$ is the effect of the *j*-th SNP marker within the *i*-th region [23].

Variance components and heritabilities were estimated by AIREMLF90. Heritability ($h^{2}$) was calculated as $h^{2}=\frac{\sigma_{a}^{2}}{\sigma_{p}^{2}}$, and the total phenotypic variance ($\sigma_{p}^{2}$) was calculated as the sum of the additive genetic variance ($\sigma_{a}^{2}$) and the residual variance ($\sigma_{e}^{2}$). 

### 2.5. Single-Step GWASs Analysis

ssGWAS is a two-step iterative procedure: (1) prediction of GEBVs using ssGBLUP, and (2) prediction of SNP effects based on GEBV. The detailed algorithm was described by Wang et al. [14].

The ssGWAS analysis was conducted independently for each trait and performed sequentially using RENUMF90 for general dataset preparation; PREGSF90 was used for quality control and generation of clean genotypes, and BLUPF90 and POSTGSF90 were used for the prediction of breeding values and SNP effects, respectively. These programs are part of the BLUPF90 software family, and for this study, the step-by-step tutorial reported by Masuda [24] was followed. 

### 2.6. Identification of Candidate Genes and Functional Enrichment Analysis

Assuming that all windows explain the same proportion of genetic variance, the proportion of genetic variance explained by each of the 2519 1 Mb windows, including the 38,268 SNPs in the sheep genome, was 0.04%. Therefore, windows that explained at least 0.22% of the genetic variance, which is 5 times higher than expected (0.045 × 5 = 0.22%), were considered to contain putative QTL [25,26]. Regions representing 0.22% or more of the genetic variance $\sigma_{a}^{2}$ were defined as significant regions. SNPs within these regions were identified and mapped onto the Oar_v3.1 sheep genome assembly using the Ensembl database [27]. A range of 5 kb upstream and downstream of the variant position was considered to identify candidate genes.

Functional enrichment analysis was conducted on the set of common candidate genes for both traits using DAVID (https://david.ncifcrf.gov/tools.jsp, accessed on 10 February 2024) [28]. Gene ontology (GO) terms with a *p*-value ≤ 0.05 were reported as significant terms.

## 3. Results

### 3.1. Variance Components and Heritabilities

The variance components and heritabilities for GIP resistance in Australian Merino sheep are presented in Table 2. The BLUP and ssGBLUP estimates of the heritabilities for FEC1 and FEC2 were close to 0.19. On the other hand, the genetic correlation between both traits was 0.88 (±0.03).

### 3.2. Genome-Wide Association Analysis

**Figure 2:**
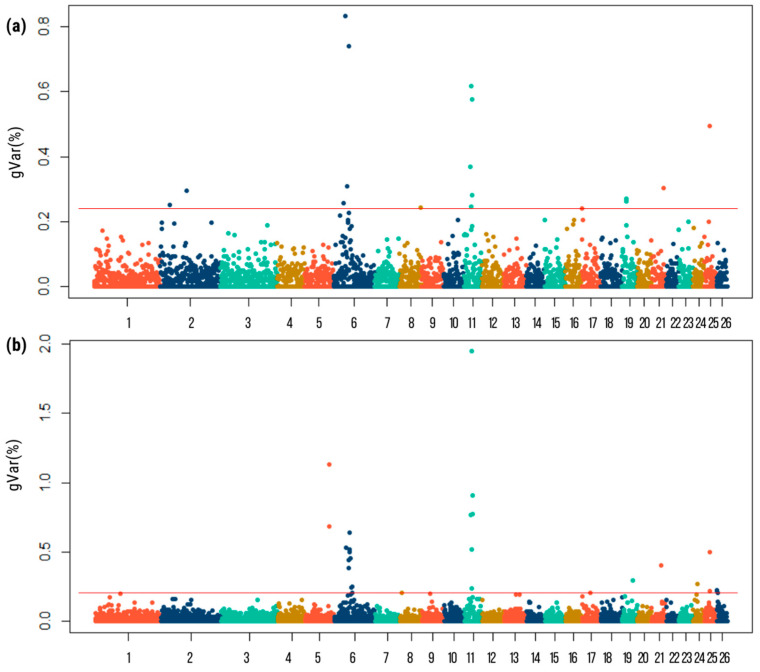
Manhattan plots depict the genetic variance explained (%) by 20 adjacent SNP windows for FEC1 (**a**) and FEC2 (**b**) in Australian Merino sheep. Each dot represents a window, with the percentage of additive genetic variance explained by each window. The horizontal line indicates the suggestive threshold of 0.22 of the gVar (%).

Figure 2 shows the Manhattan plots for GIP resistance traits. The genomic regions that explained the largest genetic variance for FEC1 were located on chromosomes 2, 6, 11, 21, and 25, and for FEC2, they were on chromosomes 5, 6, and 11. The top windows (most significant) explained 0.9% and 2% of the genetic variance for FEC1 and FEC2, respectively (Table 3).

**Figure 3:**
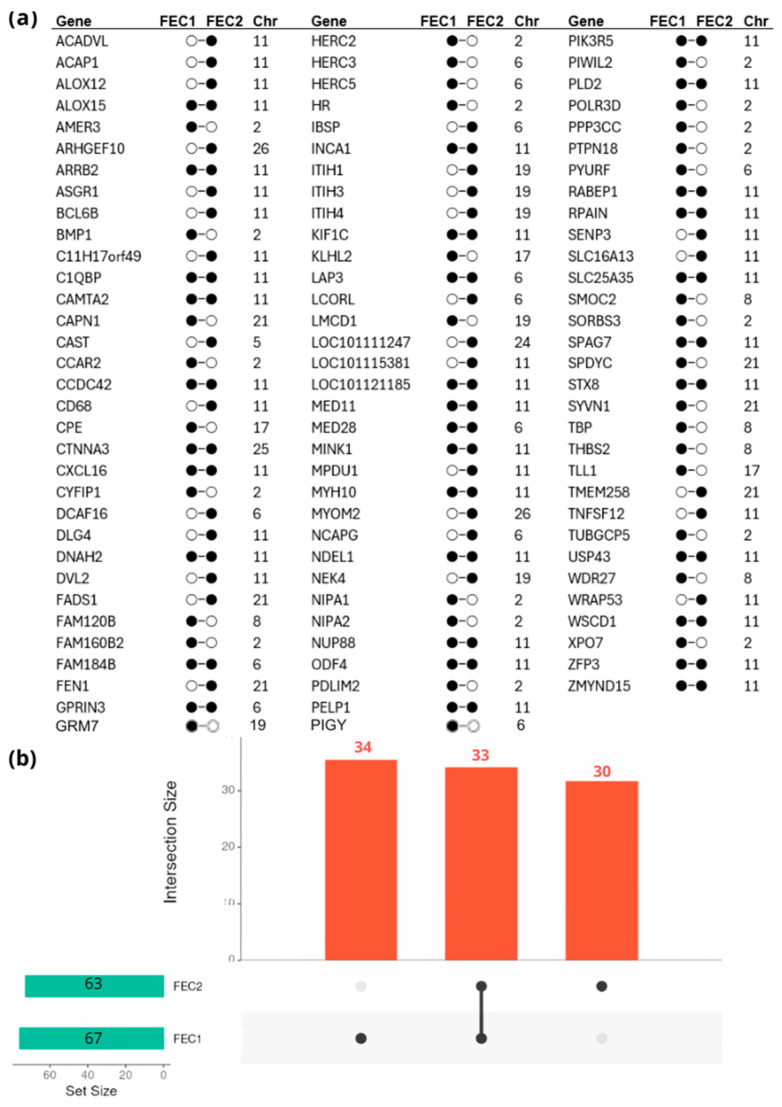
List of positional candidate genes identified in genomic regions that explain > 0.22 gVar(%) for FEC1 and FEC2 (**a**). The black dot represents that this gene was identified in the analysis for each trait. The bar plot shows the number of candidate genes in common for both traits at the intersection (**b**).

A total of 18 and 22 windows with genetic variance greater than the significance threshold of 0.22 gVar (%) were identified, which included 316 and 376 SNPs related to FEC1 and FEC2 traits, respectively. Positional candidate genes close to SNPs (≤0.5 Mbps) were identified using the *Ovis aries* 3.1. reference genome map, and a total of 67 and 63 genes were mapped for FEC1 and FEC2, respectively. In total, 33 genes were shared between both traits (Figure 3). Details of the common positional candidate genes for the FEC1 and FEC2 traits are summarized in Table 4.

### 3.3. Enrichment Analysis

**Figure 4:**
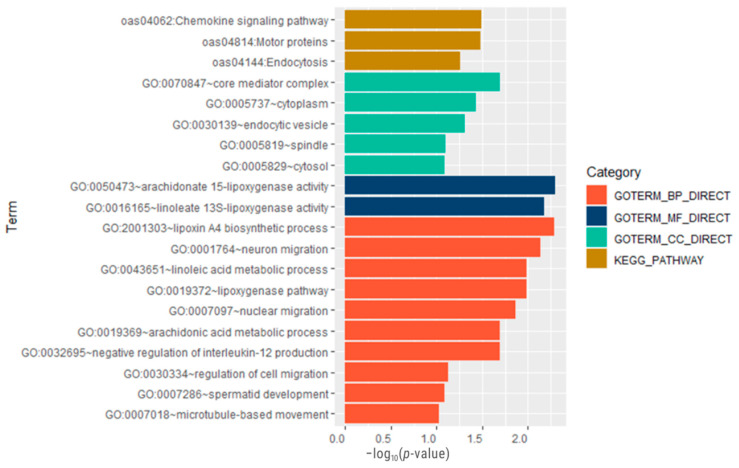
Gene ontology (GO) term enrichment analysis of genes in common associated with FEC1 and FEC2 traits in Australian Merino sheep. Categories included biological process (BP), cellular component (CC), and molecular function (MF) on the *x*-axis the -log_10_ of the adjusted *p*-value (<0.05) and on the *y*-axis the GO term.

The ssGWAS results were complemented with a gene ontology (GO) enrichment analysis, which revealed 17 significantly enriched GO terms (*p* ≤ 0.05). Among these, ten were related to biological processes, two to molecular functions, and five to cellular components. The KEGG pathway enrichment analysis revealed three enriched metabolic pathways for the set of analyzed genes (Figure 4). Details of the enriched GO categories and the metabolic pathways involved are shown in Table 5.

## 4. Discussion

Variance component estimates obtained using the traditional pedigree-based approach (BLUP) were like those obtained using the ssGBLUP procedure, as well as the estimated heritability values (0.19 vs. 0.20). Medium-to-low heritabilities for resistance to GIPs in sheep [29,30] and for Australian Merino [8] have already been reported. Genetic parameter estimates obtained using ssGBLUP are known to be less biased and more accurate [31,32,33] since relationships between animals are better estimated [34]. In our case, the results were similar, and because there were no changes in the genetic base, the same additive variance is expected when including the genomic coefficients, as reported by Forni et al. [31].

The strong genetic correlation between FEC1 and FEC2 (0.88) suggests that they can be considered to be the same trait genetically, even when these traits were measured at different ages and correspond to two different parasitic cycles and seasons of the year (Figure 1), in which animals could have been exposed to different parasites. This high estimate is in agreement with other studies that explored the genetic association between FEC recorded at different ages in different breeds (i.e., 0.85 Romney; 0.82 Katahdin) [35,36]. A high genetic correlation of 0.74 between FEC by *Strongyles* sp. and *Nematodirus* sp. was also reported by Pacheco et al. [37]. 

On the other hand, ssGWAS revealed that the genomic regions reported as significant explain only 7 and 6% of the genetic variance for FEC1 and FEC2, respectively. These small variances suggest that resistance to GIPs is a polygenic trait with a large number of variants involved in the resistance mechanism [10,38,39]. Significant regions associated with GIP resistance have been previously reported on chromosomes 2, 6, 18, and 24 in several Australian sheep populations, including the Merino breed [40], and GIP-related QTL regions are also known [41].

In this study, seventeen positional candidate genes were identified on the OAR 2 for the FEC1 trait: *AMER3*, *BMP1*, *CCAR2*, *CYFIP1*, *FAM160B2*, *HERC2*, *HR*, *NIPA1*, *NIPA2*, *PDLIM2*, *PIWIL2*, *POLR3D*, *PPP3CC*, *PTPN18*, *SORBS3*, *TUBGCP5*, and *XPO7*. Among these, several are involved in the mechanisms of innate and adaptive immunity in mammals, such as *HERC2* and *CYFIP1*, that are also involved in cytokine signaling [40,42]. The *PDLIM2* gene has been associated in transcriptomic studies with the immune system and reproduction in sheep [43]. *PTPN18* is involved in the B-cell receptor signaling pathway, being involved in differentiation, proliferation, and immunoglobulin (Ig) production [40], while other reports relate it to pigmentation in Merino sheep [44]. The *SORBS3*, *PPP3CC,* and *PIWIL2* genes have been linked to growth and wool quality traits [45]. *PPP3CC* has been associated with heat tolerance in cattle [46], while *PIWIL2* has been linked to reproductive traits in pigs [47]. In addition, these genes have been reported in selection signature studies, revealing their involvement in the parasite resistance of Slovakian sheep populations [48]. Al Kalaldeh et al. [40] also reported the association of the *HERC2*, *NIPA1*, *NIPA2*, *CYFIP1*, *TUBGCP5*, *PTPN18,* and *AMER3* genes with GIP resistance in sheep and their involvement in immune system mechanisms. In addition, the *CAST* gene (OAR 5) was identified in the ssGWAS for FEC2 and it is known to have relevance to traits such as muscle production, carcass, and meat quality in sheep [49,50]. 

On the other hand, 12 candidate genes significantly associated with the FEC1 or FEC2 traits were identified on OAR 6: *DCAF16*, *FAM184B*, *GPRIN3*, *HERC3*, *HERC5*, *IBSP*, *LAP3*, *LCORL*, *MED28*, *NCAPG*, *PIGY*, and *PYURF*; all of them except *LAP3* were detected by Al Kalaldeh et al. [40] in significant genomic regions for GIP resistance in sheep. Particularly, the *FAM184B* gene has also been reported to be strongly associated with body size and weight in livestock [51,52,53], milk production [54], and reproductive traits in sheep [55]. 

The *MED28* gene is involved in milk production in ewes [45,54] and is also related to liveweight [56]. In addition, this gene has been linked to both pre- and postnatal body weights in ewes [51,56]. On the other hand, *LAP3* also contributes to growth, milk production, and feed efficiency traits in sheep [57]. The *GPRIN3* gene has been linked to prolificacy, litter size [58], and temperament in sheep [59].

Five candidate genes were identified on OAR 8: *FAM120B*, *SMOC2*, *TBP*, *THBS2*, *WDR27*, and *WDR27*. The *THBS2* gene is related to growth and development traits [60] and high prolificacy in sheep [61], while the *WDR27* gene has been associated with the cow milk fat trait [62].

On chromosome 11, a total of 43 candidate genes for FEC1 and FEC2 were identified, including *ACADVL*, *ACAP1*, *ALOX12*, *ALOX15*, *ARRB2*, *ASGR1*, *BCL6B*, *C11H17orf49*, *C1QBP*, *AMTA2*, *CCDC42*, *CD68*, *CXCL16*, *DLG4*, *DNAH2*, *DVL2*, *INCA1*, *KIF1C*, *LOC101115381*, *LOC101121185*, *MED11*, *MINK1*, *MPDU1*, *MYH10*, *NDEL1*, *NUP88*, *ODF4*, *PELP1*, *PIK3R5*, *PLD2*, *RABEP1*, *RPAIN*, *SENP3*, *SLC16A13*, *SLC25A35*, *SPAG7*, *STX8*, *TNFSF12*, *USP43*, *WRAP53*, *WSCD1*, *ZFP3,* and *ZMYND15*. The *ALOX15* gene is known to play a role in *H. contortus* infection in sheep [63]. Other studies have reported QTLs for *T. colubriformis* egg count in the Merino breed in this genomic region [64], as well as QTLs for height, weight, and parasite resistance in the region between 32.13 and 32.19 Mbp [65]. In addition, selection signatures for resistance to *H. contortus* in sheep and goats have been reported by Estrada-Reyes et al. [66], while the CTNNA gene, located on chromosome 25, has been significantly associated with FEC1 and FEC2 traits and is related to growth traits [67] and brucellosis resistance in sheep [68]. 

The GO analysis (Figure 4) revealed multiple GO terms related to host defense mechanisms against pathogens, including the lipoxin A4 biosynthetic process (GO:2001303). Lipoxins have been reported to be endogenous anti-inflammatory molecules involved in reducing excessive tissue damage and chronic inflammation [69]. These lipoxins can be synthesized from arachidonic acid, which justifies the over-representation of biological processes such as the lipoxygenase pathway (GO:0019372) and arachidonic acid metabolic process (GO:0019369), as well as arachidonate 15-lipoxygenase activity and linoleate 13S-lipoxygenase activity molecular functions (GO:0050473, GO:001616).

Cytokines, such as IL-12, are critical for host resistance to many pathogens but can also be detrimental when expressed in an uncontrolled manner [70,71], so it makes biological sense that the GO term the negative regulation of interleukin-12 production (GO:0032695) is over-represented. The role of lipoxins in mediating the immune response has been studied against parasitic pathogens [72] and in other diseases [69]. Another GO term related to host defense against pathogens is the GO term the linoleic acid metabolic process (GO:0043651). The activation of linoleic acid metabolism in macrophages in bacteria promotes pathogen killing [73]. In that sense, one of the most over-represented genes is the *ALOX15* gene, which is related to the oxidation of arachidonic acid and the production of anti-inflammatory lipoxins [74], and it was identified as a differentially expressed gene associated with sheep resistance to the nematode *Teladorsagia circumcincta* [75].

As with all infections, parasites cause inflammation, involving the mobilization, proliferation, and recruitment of leukocytes to the affected area. This trafficking of immune cells and the effector functions of these cells can serve to control the pathogen or exacerbate the pathology [76]. In this regard, one of the most over-represented metabolic pathways is the oas04062/chemokine signaling pathway. Two of the functions of chemokines are to attract immune cells to sites of inflammation [77] and to guide the migration of neurons and other migratory cells [78].

In summary, the enrichment analysis shows that the candidate genes enriched biological processes and molecular functions related to the metabolism of linoleic acid, which is a metabolic precursor of arachidonic acid. Free arachidonic acid and its metabolites promote and modulate the type 2 immune response, playing a crucial role in GIP resistance through the action of eosinophils, basophils, and mast cells [79].

## 5. Conclusions

In this study, genetic parameters were estimated to evaluate resistance to GIPs in two independent parasite cycles, each separated by anthelmintic treatment. A total of 18 and 22 genomic regions were identified that showed a significant association with FEC1 and FEC2, respectively. We report positional candidate genes for both cycles using ssGWAS in Australian Merino sheep, some of which are novel for these traits. Our study reveals a set of candidate genes that share mechanisms related to immune response, body size, and weight, as well as genes associated with reproductive traits. In summary, our findings provide a basis for future genomic research and could contribute significantly to breeding programs.

## Figures and Tables

**Table 1:** Descriptive statistics for fecal egg counts (LogFEC1 and LogFEC2).

Trait	N	Mean	SD ^a^	Min ^b^	Max ^c^
LogFEC1	26,638	6.63	1.14	4.60	10.51
LogFEC2	18,971	6.64	1.13	4.60	10.87

^a^ SD: standard deviation; ^b^ Min: minimum; ^c^ Max: maximum.

**Table 2:** Additive genetic variances, residuals, and heritability for FEC1 and FEC2 in Australian Merino sheep.

Trait	Method	$\sigma_{a}^{2}$	$\sigma_{e}^{2}$	*h* ^2^
FEC1	BLUP	0.15 ± 0.01	0.66 ± 0.01	0.19 ± 0.01
	SSGBLUP	0.16 ± 0.01	0.66 ± 0.01	0.20 ± 0.01
FEC2	BLUP	0.15 ± 0.01	0.66 ± 0.01	0.19 ± 0.02
	SSGBLUP	0.16 ± 0.01	0.66 ± 0.01	0.19 ± 0.02

$\sigma_{a}^{2}$ = additive genetic variance; $\sigma_{e}^{2}$ = residual variance; $h^{2}$ = heritability.

**Table 3:** Chromosome, location, proportion of genetic variance, and candidate genes within the top 10 windows associated with the FEC1 and FEC2 traits in Australian Merino sheep.

Trait	Chr ^a^	gVar(%) ^b^	Windows Bounds (pb)	Candidate Genes
FEC1	6	0.83	35,429,074–35,554,875	*GPRIN3*
	6	0.73	37,072,532–37,134,706	*LAP3, MED28, FAM184B*
	11	0.61	26,119,722–26,407,016	*MINK1, PLD2, ZMYND15, CXCL16, MED11, ARRB2, PELP1, ALOX15*
	11	0.57	27,002,322–27,807,356	*DNAH2, LOC101121185, SLC25A35, ODF4, NDEL1, MYH10*
	25	0.49	21,324,145–22,654,093	*CTNNA3*
	11	0.36	25,298,833–25,633,178	*WSCD1*
	6	0.30	36,066,911–36,197,551	*HERC3, PYURF, PIGY, HERC5*
	21	0.30	42,654,067–42,734,621	*SYVN1, SPDYC, CAPN1*
	2	0.29	112,528,931–113,355,547	*HERC2, NIPA1, NIPA2, CYFIP1, TUBGCP5, PTPN18, AMER3*
	11	0.28	27,811,348–28,475,634	*CCDC42, PIK3R5, STX8, USP43*
FEC2	11	1.94	26,119,722–26,407,016	*MINK1, PLD2, ZMYND15, CXCL16, MED11, ARRB2, PELP1, ALOX15*
	5	1.13	93,463,383–93,482,724	*CAST*
	11	0.90	27,002,322–27,807,356	*DNAH2, LOC101121185, SLC25A35, ODF4, NDEL1, MYH10*
	11	0.77	27,811,348–28,475,634	*CCDC42, PIK3R5, STX8, USP43*
	11	0.76	25,298,833–25,633,178	*WSCD1*
	5	0.68	93,437,720–93,463,383	*CAST*
	6	0.64	37,072,532–37,134,706	*LAP3, MED28, FAM184B*
	6	0.53	35,429,074–35,554,875	*GPRIN3*
	11	0.52	25,744,499–26,098,771	*C1QBP, RPAIN, NUP88, RABEP1, ZFP3, KIF1C, INCA1, CAMTA2, SPAG7*
	6	0.51	37,483,582–37,665,116	-

^a^ Chr = chromosome number. ^b^ gVar(%) = proportion of genetic variance represented by each region comprising 20 SNPs.

**Table 4:** Positional candidate genes in common for FEC1 and FEC2.

Chr	Pos (bp)	Candidate Genes
6	35,511,497–37,257,065	*FAM184B, MED28, LAP3, GPRIN3*
11	25,298,833–28,475,634	*USP43, STX8, PIK3R5, CCDC42, MYH10, NDEL1, ODF4, SLC25A35, LOC101121185, DNAH2, ALOX15, PELP1, ARRB2, CXCL16, MED11, ZMYND15, PLD2, MINK1, CAMTA2, SPAG7, INCA1, KIF1C, ZFP3, RABEP1, NUP88, C1QBP, RPAIN, WSCD1*
25	22,143,113–22,654,093	*CTNNA3*

**Table 5:** Gene ontology (GO) terms such as biological processes, molecular functions, and KEGG pathways of candidate genes associated with FEC1 and FEC2 traits in Australian Merino sheep.

Category	Term	Genes	*p*-Value	−log_10_ (*p*-Value)
GOTERM_BP	GO:2001303~lipoxin A4 biosynthetic process	*ALOX15, LOC101121185*	0.005	2.289
GOTERM_BP	GO:0001764~neuron migration	*NDEL1, ENSOARG00000002682, MYH10*	0.007	2.140
GOTERM_BP	GO:0019372~lipoxygenase pathway	*ALOX15, LOC101121185*	0.010	1.989
GOTERM_BP	GO:0043651~linoleic acid metabolic process	*ALOX15, LOC101121185*	0.010	1.989
GOTERM_BP	GO:0007097~nuclear migration	*ENSOARG00000002682, MYH10*	0.014	1.865
GOTERM_BP	GO:0032695~negative regulation of interleukin-12 production	*ARRB2, C1QBP*	0.020	1.690
GOTERM_BP	GO:0019369~arachidonic acid metabolic process	*ALOX15, LOC101121185*	0.020	1.690
GOTERM_BP	GO:0030334~regulation of cell migration	*MINK1, ENSOARG00000002682*	0.075	1.128
GOTERM_BP	GO:0007286~spermatid development	*CCDC42, ZMYND15*	0.082	1.084
GOTERM_BP	GO:0007018~microtubule-based movement	*DNAH2, KIF1C*	0.093	1.029
GOTERM_CC	GO:0070847~core mediator complex	*MED11, MED28*	0.020	1.690
GOTERM_CC	GO:0005737~cytoplasm	*NDEL1, LOC101121185, CTNNA3, ENSOARG00000002682, PELP1, INCA1, ZMYND15, ENSOARG00000007066*	0.037	1.427
GOTERM_CC	GO:0030139~endocytic vesicle	*RABEP1, ARRB2*	0.049	1.313
GOTERM_CC	GO:0005819~spindle	*NDEL1, MYH10*	0.081	1.092
GOTERM_CC	GO:0005829~cytosol	*ALOX15, LOC101121185, PIK3R5, STX8, LAP3, ARRB2, C1QBP, MYH10*	0.082	1.086
GOTERM_MF	GO:0050473~arachidonate 15-lipoxygenase activity	*ALOX15, LOC101121185*	0.005	2.301
GOTERM_MF	GO:0016165~linoleate 13S-lipoxygenase activity	*ALOX15, LOC101121185*	0.007	2.176
KEGG_PATHWAY	oas04062/Chemokine signaling pathway	*PIK3R5, ARRB2, CXCL16*	0.033	1.486
KEGG_PATHWAY	oas04814/Motor proteins	*DNAH2, MYH10, KIF1C*	0.033	1.478
KEGG_PATHWAY	oas04144/Endocytosis	*RABEP1, PLD2, ARRB2*	0.055	1.260

## Data Availability

Restrictions apply to the availability of these data. Data were obtained by INIA and are available from the authors with INIA’s permission.
